# Comparing Knee Joint Load Accumulation During Cycling, Walking, and Running in Individuals With and Without Knee Osteoarthritis

**DOI:** 10.1111/sms.70369

**Published:** 2026-09-16

**Authors:** Jonas Ebbecke, Lasse Hansen, Josef Viellehner, William Brent Edwards, Nina Naeckel, Wolfgang Potthast

**Affiliations:** ^1^ Institute of Biomechanics and Orthopaedics German Sport University Cologne Cologne Germany; ^2^ Human Performance Laboratory, Faculty of Kinesiology University of Calgary Calgary Canada; ^3^ McCaig Institute for Bone and Joint Health University of Calgary Calgary Canada; ^4^ Department of Biomedical Engineering University of Calgary Calgary Canada

**Keywords:** cumulative load, cycling biomechanics, dose equivalence, knee adduction moment, knee osteoarthritis

## Abstract

Cycling is recommended as a conservative treatment for managing knee osteoarthritis (KOA), yet direct comparisons of knee joint loading between cycling and weight‐bearing locomotion are limited. This study compared fatigue‐weighted cumulative knee joint loading (*wCKL*) across cycling, walking, and running in individuals with and without KOA. Fifty‐six recreational cyclists (19 KOA, 19 age‐matched controls, 18 young controls) performed cycling at nine cadence‐power combinations (60/80/100 rpm × 157/210/261 W), walking at 1.4 m/s, and running at 3.0 m/s. Three‐dimensional kinematics and reaction forces (ground for walking/running, pedal for cycling) were used to compute external knee moments via OpenSim. *wCKL* was calculated from sagittal‐plane and frontal‐plane knee moments and scaled to 1 h of activity. Mixed‐effects linear models tested task‐ and group‐dependent effects, and dose‐equivalence estimates were derived to relate cycling duration to equivalent walking or running exposure. Across planes, cycling produced lower *wCKL* than running and was not greater than walking. In the sagittal plane, cycling and walking showed comparable *wCKL* across groups, whereas KOA exhibited reduced sagittal *wCKL* relative to controls during running. In the frontal plane, KOA demonstrated significantly elevated *wCKL* during walking and running but not cycling. Dose‐equivalence analyses indicated that cycling would need to be performed ~1–2 h to match 1 h of walking and ~3–5 h to match 1 h of running, with larger ratios in the frontal plane for KOA. These findings demonstrate that cycling limits cumulative knee loading compared with running without exceeding walking under the tested conditions.

## 1 Introduction

Knee osteoarthritis (KOA) is a leading cause of pain, disability, and joint functional decline [1, 2], with a strong relation to mechanical loading [3, 4, 5]. There is a broad scientific consensus that knee joint moments can be used as surrogate measures for joint contact forces and loading in general [3, 6, 7]. Higher sagittal‐plane knee flexion/extension moments are associated with increased muscle‐driven compressive joint forces [6, 8], while higher frontal‐plane knee adduction moments lead to a shift of contact forces toward the medial compartment. Both mechanical factors have been linked to the onset and progression of knee osteoarthritis [3, 4, 5]. Yet, maintaining aerobic activity is central to symptom management and long‐term joint health [9, 10, 11, 12, 13, 14, 15]. Accordingly, clinical guidelines from leading organizations strongly recommend aerobic exercise for individuals with KOA [16, 17, 18], and cycling is frequently used as a low‐impact modality. The overarching goal of an effective exercise program for managing KOA is therefore to promote lower extremity physical activity while minimizing joint loading. Understanding the mechanical loads that various activities impose on the knee and how these differ is essential to recommend not only a form of activity, but also the intensity and duration.

During gait, individuals with KOA typically exhibit elevated frontal‐plane loading, reflected by increased external knee adduction moment (KAM), which has been consistently linked to disease progression [3, 4]. At the same time, KOA is often associated with sagittal‐plane load avoidance strategies during weight‐bearing locomotion, including reduced external knee flexion and extension moments (KFM/KEM), increased joint stiffness, forward trunk lean, and a redistribution of sagittal‐plane loading from the knee toward the hip [19, 20, 21]. These adaptations are generally interpreted as protective strategies that reduce knee joint load under weight‐bearing conditions. Importantly, such strategies are thought to be task‐dependent and may not generalize to non‐weight‐bearing activities.

Direct comparison between varying locomotor tasks is challenging. Walking and running are weight‐bearing activities where the knee experiences relatively short but high‐magnitude cycles. Body mass, gait speed, lower limb alignment, and KOA severity are the primary determinants of knee joint loading in walking and running [22, 23]. Together, they account for the majority of the variability observed in sagittal and frontal plane knee moments during gait. By contrast, cycling is a non‐weight‐bearing activity characterized by many repetitions of longer, relatively low‐magnitude load cycles [8, 24, 25]. Under ergometer‐controlled conditions, mechanical knee joint loading during cycling is primarily influenced by the interaction between power output and cadence, while factors such as body mass, cycling speed, and lower‐limb alignment may have less direct influence than during weight‐bearing locomotion [26, 27]. These fundamental differences limit the interpretability of conventional peak‐based or body‐mass‐normalized metrics for cross‐task comparisons of knee joint loading.

Previous work has attempted to compare knee loading between cycling and weight‐bearing locomotion using cumulative load‐matching approaches. Notably, Gatti and colleagues [28] compared running and cycling by prescribing exercise bouts with equivalent cumulative external load, defined by linear integration of vertical ground reaction forces during running and vertical pedal reaction forces during cycling. Despite matched cumulative load exposure, running induced greater acute changes in knee cartilage relaxation times than cycling, highlighting task‐specific effects beyond total load magnitude alone. However, ground reaction forces do not directly reflect knee joint loading, and linear force integration does not account for the nonlinear fatigue behavior of articular cartilage, whereby higher‐magnitude loading cycles contribute disproportionately to mechanical damage. To address this limitation, cumulative knee joint loading can be modeled using fatigue‐weighted approaches that integrate load magnitude, cycle number, and duration within a nonlinear framework [29]. The present study applies such a fatigue‐weighted cumulative knee joint loading model to directly compare cycling, walking, and running in individuals with and without knee osteoarthritis.

With this study, we therefore aim to address the question: How does cumulative knee joint loading compare across cycling, walking, and running in individuals with and without KOA? Using a mechanical fatigue‐weighted model that incorporates sagittal and frontal plane kinetics [29], we quantify cumulative exposure across tasks, test whether KOA alters these patterns compared to healthy controls, and derive dose‐equivalence estimates (e.g., how much cycling equals 1 h of walking or running), including comparisons relative to energy expenditure, to facilitate the practical interpretation of mechanical knee loading across activities.

We hypothesized that

- Cumulative knee joint loading is lowest during cycling, higher in walking, and highest in running.
- Individuals with KOA exhibit reduced sagittal plane cumulative exposure during weight‐bearing locomotion (walking and running) but not cycling compared to controls.
- Individuals with KOA exhibit elevated frontal plane cumulative exposure during walking and running but not cycling compared to controls.

## 2 Methods

### 2.1 Participants

Fifty‐six participants were recruited for this study, including a group with osteoarthritis (KOA), an age‐matched control group (CO), and a young control group (CY; Table 1). This cohort is identical to that reported in our previous work [27]. All participants were recreational or competitive cyclists capable of performing the required cycling, walking, and running tasks. Participants in the KOA group were aged 40–65 years and had pre‐existing physician‐diagnosed medial tibiofemoral knee osteoarthritis (Kellgren‐Lawrence Grade 2–4, unilateral or bilateral), which was verified from medical documentation provided by the participants. They reported largely symptom‐free cycling and no additional musculoskeletal complaints at the time of testing. Age‐matched controls (CO) were aged 45–60 years, and young controls (CY) were aged 18–30 years. Participants in both control groups were excluded if they reported any musculoskeletal complaints within the previous 3 months.

**TABLE 1:** Cohort description (mean ± SD). Reproduced and extended from Ebbecke et al. [27].

	KOA	CO	CY
*N*	19	19	18
Female	4 (21%)	3 (16%)	6 (33%)
Male	15 (79%)	16 (84%)	12 (67%)
Age [years]	57.3 ± 6.7	54.1 ± 6.5	25.7 ± 6.9
Body mass [kg]	85.6 ± 17.4	76.9 ± 10.2	78.4 ± 10.1
Height [cm]	180.6 ± 9.7	180.7 ± 6.8	182.0 ± 10.0
BMI	26.1 ± 4.8	23.5 ± 2.6	23.6 ± 2.3
Marker‐based HKA [°; positive = varus]	1.53 ± 2.59	1.32 ± 2.72	0.16 ± 2.03
KL Grade 2	4 (21%)	—	—
KL Grade 3	4 (21%)	—	—
KL Grade 4	11 (58%)	—	—
Unilateral KOA	6 (32%)	—	—
Bilateral KOA	13 (68%)	—	—
Cycling Experience [years]	21.4 ± 33.3	18.4 ± 11.6	7.4 ± 6.3
Cycling Kilometers per year [km]	5915.8 ± 2823.8	6011.1 ± 1991.7	7200.0 ± 2901.5
KOOS Symptoms	74.6 ± 16.4	97.2 ± 4.2	98.7 ± 3.0
KOOS Pain	77.9 ± 17.3	98.8 ± 3.0	99.7 ± 0.9
KOOS ADL	87.8 ± 11.0	99.8 ± 0.7	99.9 ± 0.3
KOOS Sport	63.4 ± 25.9	98.3 ± 4.2	100 ± 0.0
KOOS Quality of Life	58.2 ± 22.4	97.6 ± 5.3	100 ± 0.0

Abbreviations: BMI, body mass index; CO, age‐matched control group; CY, Control Group Young; HKA, hip‐knee‐ankle angle; KL, Kellgren–Lawrence Grade; KOA, Knee Osteoarthritis Group; KOOS, Knee injury and Osteoarthritis Outcome Score.

This protocol was approved by the Ethics Committee of German Sport University Cologne (No. 211/2024) and was conducted in accordance with the Declaration of Helsinki. All participants provided written informed consent before participation.

### 2.2 Data Collection

Self‐reported knee symptoms and function were assessed using the Knee Injury and Osteoarthritis Outcome Score (KOOS; Table 1).

The cycling protocol comprised nine cadence‐power combinations spanning a submaximal range typical of recreational/sportive cycling, as described in detail in a previous publication [27]. The seat height was adjusted to match the recommendation of 25°–30° knee flexion angle at the bottom dead center [30], with the knee positioned vertically above the pedal at 90° crank angle. After a 10‐min warm‐up program with self‐selected power output and cadence, participants were asked to perform the nine cycling conditions in a randomized order. Before each recording, the target power and cadence were set in the SRMwin software, and the pedals' sensors were reset. Once the target cadence could be kept within a range of ±1 rpm, the recording was started for 30 s.

Walking and running speeds were selected to represent commonly used steady‐state gait and running conditions in biomechanics research. For the gait trials, the subjects were asked to repeatedly walk/run within a defined velocity range (level‐walking: 1.4 ± 0.1 m/s, running: 3.0 ± 0.1 m/s) until 5 valid trials were reached. A trial was counted as valid if the subject met the velocity criterion and had a complete, single foot contact on the force plate with no visually identified targeting. All data were collected within the same experimental session.

### 2.3 Motion Capture and Inverse Dynamics

Lower body kinematics were measured using a 16‐camera motion capture system (Qualisys AB, Gothenburg, Sweden; 200 Hz) and the CAST lower body marker set [31]. 3D pedal reaction forces were measured using custom‐made instrumented cycling pedals [32], and 3D ground reaction forces were measured using flush‐mounted force plates (Kistler Instrumente AG, Winterthur, Switzerland), both at 2000 Hz. Inverse kinematics and dynamics calculations were carried out using musculoskeletal modeling in OpenSim 4.5 [33]. The model proposed by Catelli et al. [34] was extended to three rotational degrees of freedom around the knee joint to allow computation of three‐dimensional external knee moments. It was scaled non‐uniformly based on participant‐specific anatomical landmark markers and virtual joint centers [35, 36]. Marker‐based Hip‐Knee‐Ankle angle (HKA) was calculated from the hip, knee, and ankle joint centers during the static calibration trial, with positive values indicating varus alignment. Kinematic and kinetic data were filtered using a zero‐lag 4th‐order low‐pass Butterworth filter with a cut‐off frequency of 10 Hz. Motion capture, force measurements, musculoskeletal modeling, and inverse dynamics procedures were identical to those described previously [27].

### 2.4 Weighted Cumulative Knee Loading

To quantify cumulative knee joint loading, we applied the fatigue‐weighted cumulative knee loading (*wCKL*) framework previously described in detail by Ebbecke et al. [27]. Briefly, the method is inspired by the Palmgren‐Miner fatigue framework [29, 37] and applies nonlinear magnitude weighting to external knee joint moment‐time series:

(1) $$\text{wCKL}=n{\left[\int_{t_{i}}^{t_{f}}M{\left(t\right)}^{b}\mathrm{dt}\right]}^{1/b}$$

Here, *wCKL* was calculated separately for the frontal plane (*wCKL*_*FP*_) and sagittal plane (*wCKL*_*SP*_) moments. Moment‐time curves were first averaged across cycles for each condition, raised to the power *b*, integrated over one cycle, and transformed back by taking the *1/b* root. Here, *b* refers to a tissue‐specific weighting factor, which was adopted as *b* = 12.9, following Miller and Krupenevich [38] who fit the power law to tibiofemoral cartilage data [39]. Because *b* is known to vary with tissue type and testing conditions, dose‐equivalence estimates were derived as a range from *b* = 12.9 ± 4 to add robustness with respect to the choice of exponent. Finally, transformed weighted moment integrals were scaled by the number of cycles (*n*) performed during 1 h of activity. The cycle count was intentionally placed outside the root so that repeated identical cycles contribute linearly to the resulting cumulative exposure metric. Accordingly, *wCKL* should be interpreted as a fatigue‐weighted exposure metric inspired by the Palmgren‐Miner framework rather than as a direct measure of tissue damage. The resulting dimensional unit is *Nm·s* ^*1/b*^, with values normalized to 1 h of activity.

*wCKL*_*FP*_ was calculated from the absolute value of the external knee adduction (*KAM*) portion of the frontal‐plane moment curve only, with the knee abduction portion set to zero. In contrast, *wCKL*_*SP*_ was calculated from the rectified sagittal‐plane moment curve by taking the absolute value of the complete curve, thereby including both external knee flexion (*KFM*) and extension moments (*KEM*) irrespective of moment direction. For each condition, moment‐time curves were segmented into complete crank revolutions or gait cycles, respectively, and mean cycle curves were calculated to represent steady‐state loading patterns. To obtain cumulative values normalized to 1 h of each locomotion task, the *wCKL* measures were scaled by the number of cycles corresponding to the subject‐ and task‐specific cadence (cycling) or stride frequency (walking/running). *wCKL*_*FP*_ and *wCKL*_*SP*_ were calculated for the affected limb, or if both legs were affected, for the more symptomatic limb as self‐reported in KOA. In both control groups, metrics were calculated for a randomly selected limb.

### 2.5 Energy Expenditure

Additionally, energy expenditure was estimated using MET values from literature [40] and the mean body mass of the KOA group (85.6 kg) as gross energy expenditure (kcal = MET × body mass [kg] × duration [h]). MET values were selected to match the experimental conditions: 3.8 for walking at 1.4 m/s, 10.5 for running at 3.0 m/s, and 10.8 for stationary cycling at 210 W. Cycling energy expenditure was calculated for the dose‐equivalent durations derived for *b* = 12.9 ± 4, with the reported ranges spanning the corresponding minimum and maximum estimates across both loading planes. Relative knee loading at equal energy expenditure was calculated from the ratio of cycling duration for equal energy expenditure to the cycling duration required for equivalent *wCKL*.

### 2.6 Statistics

Cycling data were first collapsed within each subject by averaging across the nine cadence‐power conditions, resulting in one cycling value per subject per plane. This approach was chosen because the primary aim was to characterize general differences in cumulative loading between locomotor tasks rather than differences specific to a particular cycling workload. All predefined cycling conditions were therefore weighted equally to summarize loading across the complete experimentally tested submaximal range without prioritizing a specific cadence‐power combination. This ensured a balanced comparison with walking and running, which were each assessed under a single standardized condition. To assess whether the cross‐task findings depended on this averaging approach, the cross‐task analyses were additionally repeated using the central cycling condition (80 rpm, 210 W) only; this sensitivity analysis yielded the same statistical conclusions (Appendices S5 and S6).

Mixed‐effects linear models (MLMs) were used for all analyses to account for repeated measures, nested data structure, and interindividual variability. Separate models were fit for *wCKL*_*SP*_ and *wCKL*_*FP*_. Each model included locomotion type (cycling, walking, and running), group (KOA, CO, and CY), and their interaction as fixed effects, with body mass and sex as covariates. Subject‐specific random intercepts were specified to account for within‐subject dependency. Model‐based estimated marginal means (EMMs) were computed for each group × task combination at the mean body mass and the modal sex of the study sample.

Post hoc pairwise contrasts were restricted to between‐group comparisons within a task and between‐task comparisons within a group, to explicitly address the study hypotheses. For each contrast, differences in EMMs (ΔEMM) were estimated together with their standard errors, Wald *z*‐tests, and 95% confidence intervals, using the covariance matrix of model coefficients. To control the family‐wise error rate, Holm correction was applied across the reduced set of contrasts.

Given the reduced model complexity and the large expected task‐related effects [27], the available sample size was considered sufficient for the present cross‐task comparisons.

All MLM analyses were performed using the statsmodels [41] (version 0.13.5), and pingouin [42] (version 0.5.5) packages in Python (version 3.9.18).

## 3 Results

### 3.1 Differences Between Locomotion Tasks

#### 3.1.1 Sagittal Plane

**FIGURE 1:**
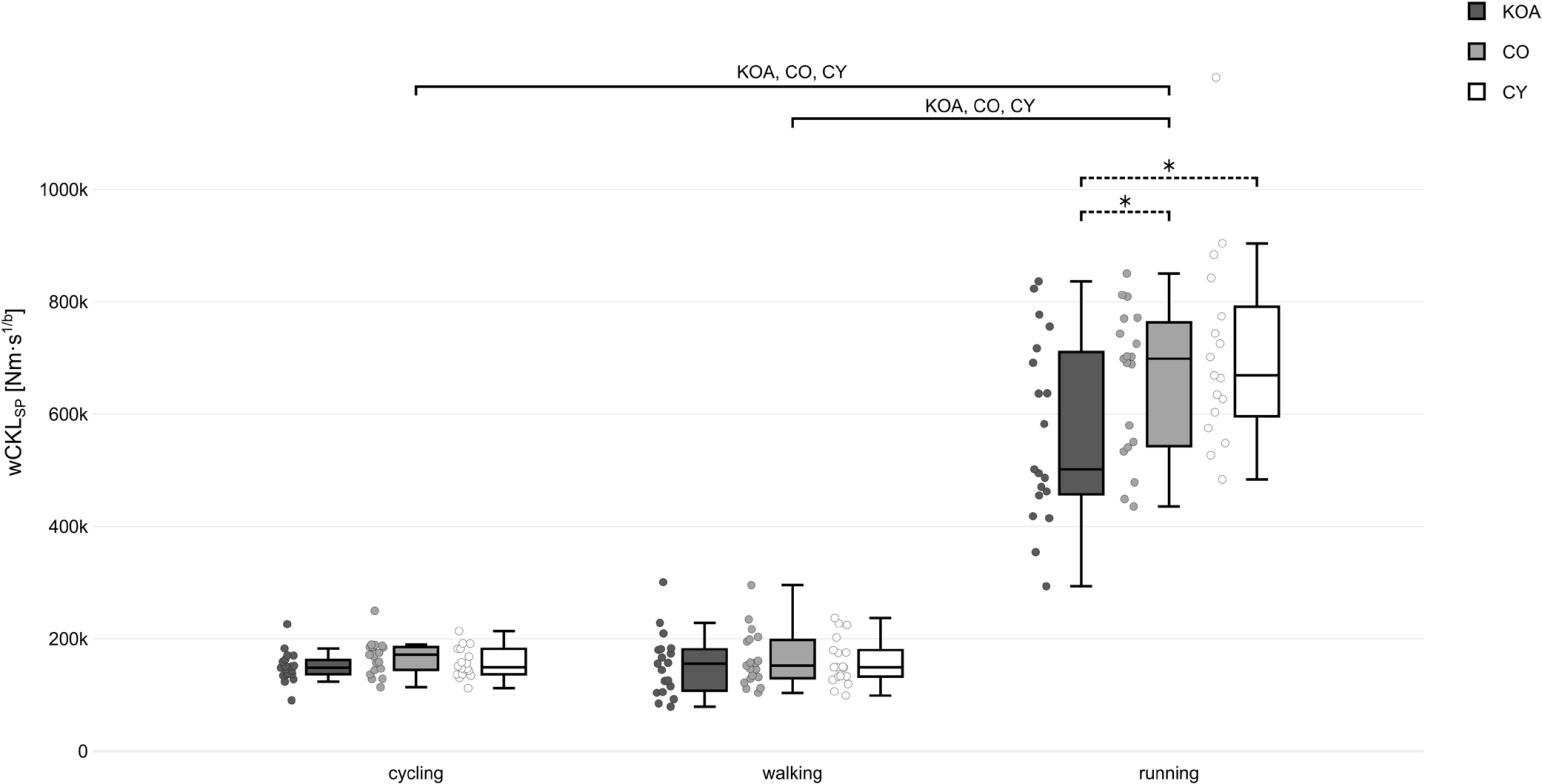
Boxplots of weighted cumulative sagittal‐plane knee joint loading (wCKL_SP_) for each group and locomotion task. Cycling trials were collapsed to a single value per subject by averaging wCKL_SP_ across all cycling conditions, ensuring a balanced within‐subject design. Solid brackets indicate significant differences between locomotion tasks, with group labels above brackets denoting the groups for which the task effect was significant. Dashed brackets with asterisks indicate significant between‐group differences within a locomotion task. All significance markers denote Holm‐adjusted post hoc tests with *p* < 0.05.

Sagittal plane task comparisons are visualized in Figure 1. The fitted MLM converged successfully. Relative to cycling, running was associated with substantially greater cumulative loading (*β* = 418 901 Nm·s^1/b^, SE = 26 575, *p* < 0.001), whereas walking did not differ significantly from cycling (*β* = 3207, SE = 26 575, *p* = 0.904). Body mass was a significant positive covariate (*β* = 3034 Nm·s^1/b^ per kg, SE = 671, *p* < 0.001). Significant task × group interactions were observed for the CY group during running (*p* = 0.001), indicating greater sagittal loading compared to the reference group. A trend toward higher loading in CO vs. KOA during running was also observed (*p* = 0.052).

Post hoc comparisons based on estimated marginal means (Appendix S1) confirmed that cycling and walking produced 73.5%–76.7% lower sagittal cumulative loading than running across all groups (all *p* < 0.001, g = 2.6–4.4, large effects). In contrast, walking and cycling did not differ in any group (all *p* > 0.90). During running, sagittal *wCKL*_*SP*_ was 17.3%–23.5% lower in KOA compared with both CO (ΔEMM = 116 095 Nm·s^1/b^, *p* < 0.001, g = 0.60) and CY (ΔEMM = 170 342 Nm·s^1/b^, *p* < 0.001, g = 0.83), corresponding to medium‐to‐large effects. No differences were observed between CO and CY during running. Thus, sagittal‐plane differences between groups emerged only during running as the highest‐load locomotor task.

#### 3.1.2 Frontal Plane

**FIGURE 2:**
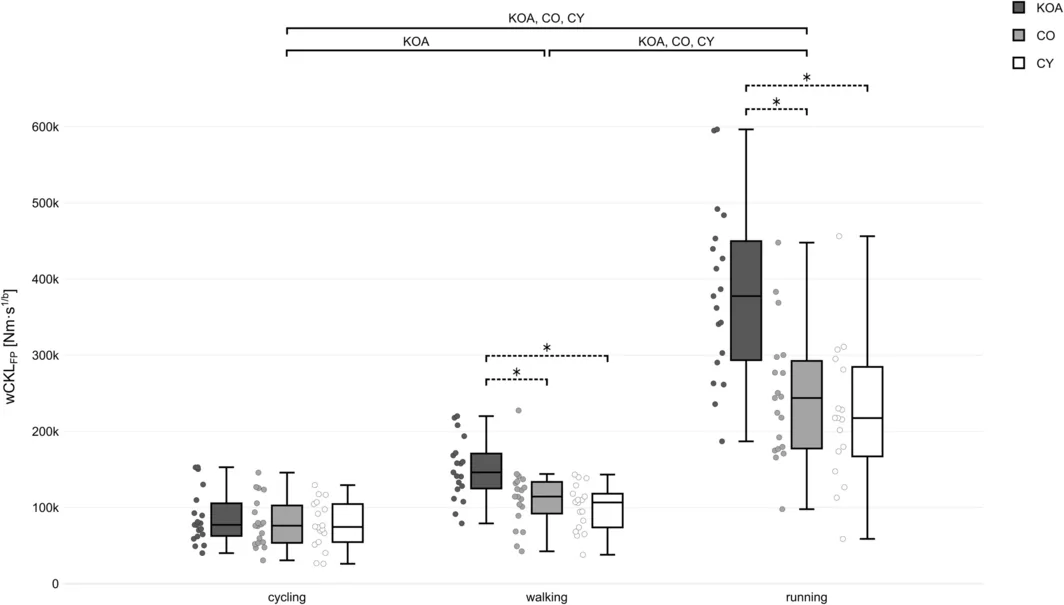
Boxplots of weighted cumulative frontal‐plane knee joint loading (wCKL_FP_) for each group and locomotion task. Cycling trials were collapsed to a single value per subject by averaging wCKL_FP_ across all cycling conditions, ensuring a balanced within‐subject design. Solid brackets indicate significant differences between locomotion tasks, with group labels above brackets denoting the groups for which the task effect was significant. Dashed brackets with asterisks indicate significant between‐group differences within a locomotion task. All significance markers denote Holm‐adjusted post hoc tests with *p* < 0.05.

Frontal plane task comparisons are visualized in Figure 2. The MLM converged successfully. Relative to cycling, both walking (β = 63 080 Nm·s^1/b^, SE = 16 555, *p* < 0.001) and running (*β* = 294 264 Nm·s^1/b^, SE = 16 555, *p* < 0.001) were associated with significantly greater *wCKL*_*FP*_. Body mass again emerged as a positive covariate (*β* = 1918 Nm·s^1/b^ per kg, SE = 487, *p* < 0.001). No significant main effects of group were observed. However, a significant task × group interaction indicated that the increase in frontal‐plane cumulative loading from cycling to walking and running was larger in KOA than in both control groups.

Post hoc within‐group comparisons (Appendix S2) confirmed, in KOA participants, cycling was associated with 44.5% lower *wCKL*_*FP*_ than walking (ΔEMM = 63 080 Nm·s^1/b^, *p* = 0.001, g = 1.19), while walking was associated with 62.0% lower *wCKL*_*FP*_ than running (ΔEMM = 231 184 Nm·s^1/b^, *p* < 0.001, g = 2.83). In both CO and CY, however, walking loads were not significantly different compared to cycling. In contrast, both cycling and walking were associated with 52.7%–78.9% lower *wCKL*_*FP*_ than running (all *p* < 0.001), with large effect sizes across groups (g = 1.6–2.8).

Between‐group comparisons revealed no significant differences during cycling. During walking and running, however, KOA participants demonstrated 17.8%–62.0% greater *wCKL*_*FP*_ than both CO and CY (all *p* < 0.01, |g| = 0.9–1.5), indicating large group effects. No differences were detected between CO and CY in any condition. Thus, disease‐specific elevations in cumulative frontal‐plane loading emerged exclusively during weight‐bearing locomotion.

### 3.2 Dose‐Equivalence Estimates

To estimate the relative mechanical loading equivalence between cycling and weight‐bearing locomotion, *wCKL*_*SP*_ and *wCKL*_*FP*_ were used to derive dose‐equivalence estimates, expressed as the cycling duration required to match 1 h of walking at 1.4 m/s or running at 3.0 m/s.

**FIGURE 3:**
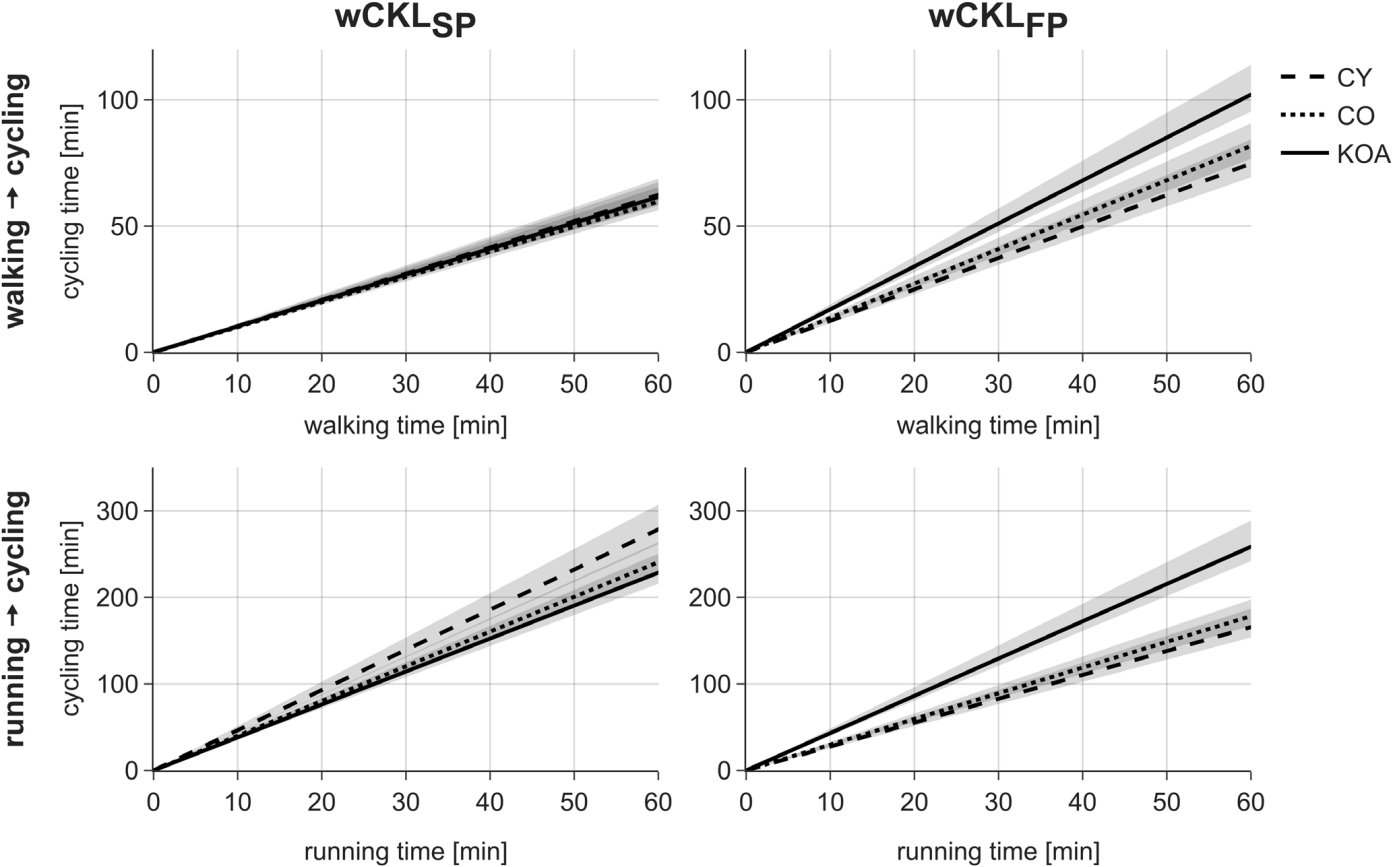
Dose‐equivalence between cycling and locomotion for knee joint loading. Lines map the duration of steady‐state cycling (80 rpm, 210 W) required to produce an equivalent cumulative knee joint load as a given duration of locomotion. The top row shows running at 3.0 m/s, and the bottom row shows walking at 1.4 m/s. The left column represents sagittal‐plane weighted cumulative knee load (wCKL_SP_), and the right column represents frontal‐plane weighted cumulative knee load (wCKL_FP_). Solid, dotted, and dashed lines represent group‐averaged equivalence relations for participants with knee osteoarthritis (KOA), age‐matched, older controls (CO), and younger controls (CY), respectively. Shaded bands indicate uncertainty in the weighting factor *b* = 12.9 ± 4 used in the weighted cumulative load model.

The comparisons were based on a range of exponent values of *b = 12.9 ± 4* used in the loading model, reflecting uncertainty in the nonlinear weighting of load magnitude. Exemplary results for cycling at 210 W and 80 rpm, walking, and running are shown in Figure 3. For illustration, cycling at 210 W and 80 rpm in KOA participants was estimated to require approximately 53–120 min to match the cumulative loading of 1 h of walking, and 194–307 min to match 1 h of running, depending on the weighting exponent and loading plane considered. Comparable patterns were observed in the CO and CY groups, with running consistently producing the highest dose equivalents. In the frontal plane, dose‐equivalence estimates relative to walking and running were considerably higher in KOA than in CO and CY, whereas in the sagittal plane, running‐related equivalence estimates were lower in KOA than in CY but similar to CO. The full set of conversion estimates for all cycling conditions is reported in Appendices S3 and S4.

When accounting for energy expenditure, 1 h of walking and running corresponded to approximately 330 kcal and 900 kcal, respectively. Cycling at 80 rpm and 210 W required approximately 820–1850 kcal to accumulate an equivalent knee load to 1 h of walking and 2990–4730 kcal to match 1 h of running. Conversely, at equivalent energy expenditure, cycling resulted in approximately 18%–40% and 19%–30% of the cumulative knee loading of walking and running, respectively.

## 4 Discussion

This study investigated mechanical fatigue‐weighted cumulative knee joint loading in both the sagittal and frontal planes across cycling, walking, and running in individuals with and without KOA. The present findings largely support the established hypotheses, with distinct patterns emerging across locomotor tasks and loading planes.

### 4.1 Task‐ and Group‐Dependent Differences in Cumulative Knee Loading

The key result of this study is that cumulative loads during cycling were consistently lower than those during running and not greater than those during walking under the conditions tested. Running, by contrast, imposed the greatest sagittal and frontal loading across all groups. The pattern of differences between cycling and walking depended on the plane of motion and group affiliation, with walking exceeding cycling only in the frontal plane among participants with KOA, but not the healthy controls.

#### 4.1.1 Sagittal Plane

In the sagittal plane, cumulative loading of cycling and walking was largely comparable across all groups, while running resulted in significantly higher cumulative loads. This pattern is consistent with the known role of knee flexion and extension moments in muscle‐driven compressive loading. Walking at a moderate speed generates relatively moderate sagittal moments, while running is characterized by increased quadriceps activity and higher vertical ground reaction forces, which increase sagittal moments and thereby compressive loading on the tibiofemoral joint [43, 44, 45, 46]. As hypothesized, individuals with KOA demonstrated reduced sagittal loading during running compared with controls. This finding is consistent with previously described sagittal‐plane unloading strategies in KOA, in which knee joint moments are reduced, and loading is redistributed toward the hip joint [19, 20, 21]. Notably, in the present study, these alterations were observed only during running, but not during walking. This discrepancy may reflect the high functional status and activity level of our KOA cohort. Despite radiographic disease (KL Grade 2–4), participants with KOA reported relatively high self‐reported function and low symptom burden during daily activities, as indicated by KOOS ADL scores averaging 87.8 ± 11.0 and KOOS Pain scores of 77.9 ± 17.3 (Table 1). In contrast, more demanding activities were rated substantially lower (KOOS Sport: 63.4 ± 25.9), suggesting that functional limitations became apparent primarily under higher mechanical demands. Walking at a moderate speed may therefore not have exceeded the functional threshold required to elicit compensatory sagittal‐plane unloading strategies in this cohort, whereas running provoked protective adaptations typically observed in KOA. This pattern is consistent with previous reports showing that gait alterations are more pronounced in individuals with more advanced disease or greater symptom severity, and may be less evident in active or early‐stage KOA populations [19, 47].

Taken together, these findings provide only partial support for hypothesis (b): individuals with KOA exhibited reduced sagittal cumulative exposure during running, but not during walking or cycling, compared with healthy controls.

#### 4.1.2 Frontal Plane

In contrast to the sagittal plane, hypothesis (c) was fully supported in the frontal plane: individuals with KOA exhibited elevated cumulative exposure during walking and running, but not cycling, compared with healthy controls. Whereas frontal‐plane loads during cycling were comparable across groups, KOA participants showed significantly greater cumulative loading than both age‐matched and young controls during walking and running, with large effect sizes. The absence of differences between CO and CY confirms that these elevations are disease‐specific rather than age‐related. Running further amplified these disease‐specific differences, resulting in the largest KOA‐control contrasts observed in this study, likely due to higher peak adduction moments combined with greater cycle counts. This pattern parallels a large body of literature on peak KAM, where KOA patients consistently demonstrate greater peak KAM values in gait compared with healthy individuals [3, 4], and extends these findings to cumulative, fatigue‐weighted loading.

When comparing tasks within groups, cycling imposed the lowest cumulative frontal‐plane loads in KOA, followed by walking and running. In the control groups, by contrast, walking and cycling did not differ significantly, further emphasizing that the additional frontal‐plane load during walking and running emerges primarily in the presence of disease. Together, these results highlight that disease‐related elevations in frontal‐plane loading manifest only during weight‐bearing tasks, while no between‐group differences were observed during cycling.

#### 4.1.3 Synthesis Across Planes

Overall, the findings provided partial support for Hypothesis (a): cumulative knee joint loading was lowest during cycling, intermediate during walking, and highest during running in the KOA group only. The cycling‐walking contrast, however, depended on the plane of motion and group affiliation, with differences emerging only in the frontal plane for participants with KOA. This pattern suggests that while compressive forces induced by sagittal moments remain broadly comparable between cycling and walking, frontal‐plane loading reveals disease‐specific mediolateral shifts that amplify cumulative exposure in KOA during weight‐bearing tasks.

### 4.2 Clinical Implications and Practical Translation

To our knowledge, this is the first study to directly compare knee joint loading between weight‐bearing (walking and running) and non‐weight‐bearing (cycling) locomotion using a fatigue‐weighted cumulative load model. The magnitude of the observed differences underscores the potential role of cycling for limiting cumulative knee loading, particularly for individuals with KOA. To facilitate practical translation, we derived dose‐equivalence estimates that relate cycling duration to equivalent cumulative knee loading during walking and running.

Our dose‐equivalence results align directionally with previous work comparing cycling and running. Gatti et al. [28] reported that cycling required substantially longer durations than running to achieve matched cumulative external load when load exposure was quantified by linear integration of vertical reaction forces, with approximately 15 min of running corresponding to ~46 min of cycling. Despite this matching, running induced greater acute changes in knee cartilage relaxation times than cycling, highlighting task‐specific effects beyond total linearly accumulated load. Using the *wCKL* metric based on the Palmgren‐Miner rule [29, 37], the present results yield comparable but slightly higher equivalence estimates, indicating that cycling would need to be performed approximately 3–5 times longer than running to achieve equivalent cumulative knee loading, depending on loading plane, disease status, and model exponent. This modest upward shift is consistent with the nonlinear weighting of load magnitude inherent to fatigue‐based models, although direct comparison between studies is complicated by differences in study populations, experimental protocols, and loading metrics. Within the present framework, equivalence estimates were particularly elevated in the frontal plane for KOA, consistent with disease‐specific amplification of medial compartment loading during weight‐bearing tasks. Compared to Gatti et al. [28], the present approach extends prior work by translating externally measured ground reaction forces to joint‐level loading and by incorporating fatigue‐based accumulation rather than linear summation. While linear integration of ground reaction forces captures the overall directional relationship between tasks, it does not account for the nonlinear weighting of load magnitude to tissue fatigue behavior. In contrast, the fatigue‐weighted cumulative knee joint loading framework applied here captures the combined influence of load magnitude, duration, and cycle number on cumulative mechanical exposure.

From a clinical perspective, the practical implication is clear: cycling imposes substantially lower cumulative knee joint loading per unit time than walking or running for individuals with KOA. When cycling at 80 rpm and 210 W, individuals with KOA would need to cycle approximately 1–2 h to match the cumulative knee loading of 1 h of walking at 1.4 m/s, and approximately 3–5 h to match 1 h of running at 3.0 m/s. This advantage becomes particularly apparent when knee loading is considered relative to energy expenditure. For an equivalent energy expenditure, cycling at 80 rpm and 210 W produced only approximately 18%–40% of the cumulative knee loading observed during walking and 19%–30% of that observed during running. Comparable trends were observed in the healthy control groups. Thus, cycling combines substantial energetic demand with considerably lower cumulative mechanical knee exposure than weight‐bearing locomotion. These characteristics may help explain why cycling is frequently associated with symptom relief and improved function in KOA. The combination of low cumulative mechanical loading and substantial aerobic demand may promote cartilage health [12, 13] and mobility [9] through repetitive joint motion and improved nutrient supply. At the same time, elevated energy expenditure supports weight management, thereby further reducing knee loading during daily activities [15].

### 4.3 Limitations

This study has several limitations. First, the experimental protocol relied on controlled conditions (constant cadence and power during cycling, fixed velocities for walking and running), which may not reflect real‐world variability. Second, participants were exclusively active cyclists, limiting generalizability to less active individuals with KOA. The presented results may not generalize to more symptomatic patients, patients unable to run, non‐cyclists, or individuals beginning cycling rehabilitation. Third, although the *wCKL* framework was originally developed to describe the accumulation of repetitive stress and strain, it was applied here to external knee joint moments. Accordingly, the cartilage‐derived weighting exponent of *b* = 12.9 has not been validated as a tissue‐specific fatigue exponent for external knee joint moments, and *wCKL* should therefore be interpreted as a nonlinearly weighted mechanical exposure index rather than a quantitative estimate of tissue fatigue or damage. While sagittal‐ and frontal‐plane knee moments are well‐established surrogate measures of compressive loading and medial load distribution, respectively, they do not directly quantify tibiofemoral joint contact forces or tissue‐level stress. Future studies could therefore extend the present framework by applying fatigue‐weighted load accumulation to subject‐specific estimates of knee joint contact forces derived from musculoskeletal modeling or to tissue‐level stress and strain estimated using finite element analyses. The presented framework also assumes that cumulative loading of biological tissues follows fatigue principles analogous to those described by the Palmgren‐Miner rule [29, 37]. Although experimental evidence supports fatigue behavior in bone [48], tendon [49], and articular cartilage [38], the precise damage accumulation characteristics of living tissues remain incompletely understood. Finally, the present study focused exclusively on mechanical loading metrics and did not assess concurrent clinical symptoms, biological responses (e.g., cartilage biomarkers or imaging outcomes), or longitudinal disease progression. As a result, the dose‐equivalence estimates should be interpreted as mechanical rather than clinical equivalences, and future longitudinal or interventional studies are needed to establish direct links between cumulative mechanical loading, symptoms, and structural disease outcomes in KOA.

## 5 Conclusion

Using a fatigue‐weighted cumulative loading framework, this study provides a direct mechanical comparison of knee joint loading across cycling, walking, and running in individuals with and without knee osteoarthritis. Cycling produced lower cumulative loading than running and was not greater than walking under the tested conditions. Disease‐specific effects were task‐ and plane‐dependent: individuals with KOA exhibited reduced sagittal‐plane cumulative loading during running, consistent with protective unloading strategies, but significantly elevated frontal‐plane loading during weight‐bearing locomotion. These differences were absent during cycling.

Dose‐equivalence estimates showed that substantially longer cycling durations are required to match the cumulative knee loading of walking or running, particularly in the frontal plane and in KOA. Together with the lower loading observed for equivalent energy expenditure, these findings demonstrate that cycling can provide substantial aerobic activity while limiting cumulative mechanical knee exposure and provide a mechanical basis for its use as a low‐impact exercise modality in individuals with KOA.

## 6 Perspectives

This study provides a mechanical framework to compare knee joint loading across locomotor tasks, but the clinical relevance ultimately depends on the biological response to loading. Future research should therefore focus on the dose–response relationship between cumulative mechanical exposure and joint health in knee osteoarthritis. Mechanical load alone does not determine outcomes; factors such as cartilage metabolism, inflammation, and tissue adaptation influence whether loading is beneficial or harmful. Longitudinal studies integrating biomechanical metrics with imaging, biochemical markers, and symptom progression are needed to identify individualized loading thresholds and to better understand how different activity types contribute to joint health over time.

## Funding

This project was funded by the Internal Research Funds of the German Sport University Cologne, grant agreement number L‐11‐10011‐283‐093000. The funding source had no involvement in the study design, data collection, analysis, or interpretation, writing of the report, the decision to submit the article for publication, or any other aspect of the research.

## Conflicts of Interest

The authors declare no conflicts of interest.

## Supporting information


**Appendix S1:** Post hoc test results and EMMs of wCKL_SP_ for each group and condition combination.

**Appendix S2:** Post hoc test results and EMMs of wCKL_FP_ for each group and condition combination.

**Appendix S3:** Conversion estimates from each cycling condition and group to walking at 1.4 m/s and running at 3 m/s based on wCKL_SP_ and exponent range b = 12.9 ± 4.

**Appendix S4:** Conversion estimates from each cycling condition and group to walking at 1.4 m/s and running at 3 m/s based on wCKL_FP_ and exponent range b = 12.9 ± 4.

**Appendix S5:** Sensitivity analysis of sagittal‐plane weighted cumulative knee loading (wCKL_SP_) using the central cycling condition (80 rpm, 210 W).

**Appendix S6:** Sensitivity analysis of frontal‐plane weighted cumulative knee loading (wCKL_FP_) using the central cycling condition (80 rpm, 210 W).

## Data Availability

The data of this study are publicly available in the Open Science Framework (OSF) at https://doi.org/10.17605/OSF.IO/97QYW.
